# Effect of Carbon Dioxide’s Injection Concentration on the Diffusion Coefficient of Methane

**DOI:** 10.1021/acsomega.5c10759

**Published:** 2025-12-08

**Authors:** Jianchi Hao, Shuangming Wang, Hu Wen, Zegong Liu, Lifeng Ren

**Affiliations:** † College of Geology and Environment, Xi’an University of Science and Technology, Xi’an 710054, Shaanxi, China; ‡ College of Safety Science and Engineering, Xi’an University of Science and Technology, Xi’an 710054, Shaanxi, China; § College of Safety Science and Engineering, Anhui University of Science and Technology, Huainan 232001, Anhui, China

## Abstract

The process of displacing methane (CH_4_) using carbon dioxide (CO_2_) in coal seams involves the adsorption and diffusion of a binary gas mixture in coal. Diffusion serves as a bridge linking adsorption, desorption, and seepage of the two gases, thereby affecting the production of coal-bed methane (CBM) and the injection of CO_2_. In the present paper, the adsorption, desorption, and diffusion behaviors of both gases were investigated during the displacement of CH_4_ using CO_2_ in coal seams. The results indicate that the CO_2_ injection process to enhance CH_4_ drainage from coal seams can be divided into three stages: a stage where both replacement and displacement effects occur simultaneously; a stage dominated by replacement effects; and a steady state. Based on the fitting relationships of the diffusion amounts of CH_4_ and CO_2_ gases with experimental time, computational models for the diffusion coefficients of both gases were established. The calculation results indicate that the diffusion coefficients for both gases decrease rapidly with an increase in experimental time. Furthermore, as the injection concentration of CO_2_ increases, the diffusion coefficient of CH_4_ shows an upward trend, while the diffusion coefficient of CO_2_ exhibits a downward trend.

## Graphical abstract

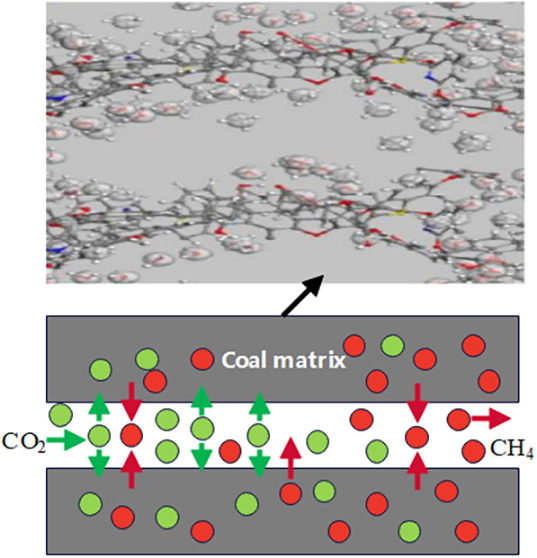

## 1 Introduction

Coal is the fundamental and most-used source of energy in China. As an efficient and clean resource associated with coal, methane’s efficient extraction is the fundamental method for preventing and controlling methane-related disasters and accidents, as well as obtaining clean energy.^1,2^ China’s coal seam structure is complex, and coal permeability is generally low. As shallow coal resources become increasingly depleted, the development of coal has shifted to deeper levels. The low permeability characteristics of coal seams have become increasingly evident, making the extraction of methane more difficult and increasing the pressure on preventing and controlling methane-related disasters.^3,4^ Traditional negative pressure extraction technology cannot overcome the challenge of low permeability in coal seams. Pre-extraction takes a long time and yields slow results, failing to meet the needs of postmining succession and safe underground production.^5^ To address the challenge of low permeability in coal seams, China has implemented various measures such as mining protective layers, hydraulic fracturing/slotting/punching, and loosening blasting to enhance the drainage of methane and improve the permeability of coal seams.^6^ Although most permeability enhancement measures successfully improve the permeability of coal seams, the problem of a sharp decline in extraction efficiency due to the decrease in reservoir pressure during the later stages of mining remains unresolved. There is an urgent need for simple and effective technology to improve the permeability of coal seams and enhance the extraction efficiency of gas.^7–9^ With the continuous advancement of science and technology, breakthroughs have been made in gas injection displacement-replacement technology.^10–12^ The successful experiments of CO_2_-enhanced coalbed methane (CO_2_-ECBM) have provided a novel idea for using gas injection displacement to improve the extraction efficiency of underground gas.^13–15^ By injecting CO_2_ into coal seam, it can not only compete with methane (CH_4_) stored in the coal seam for adsorption, reduce the effective partial pressure of gas, and promote the desorption of adsorbed gas, but can also increase the internal pressure of the coal body, enhance the mixed gas percolation velocity, effectively compensate for the reservoir pressure drop during the later stages of extraction, and provide sufficient power and reliable migration pathways for the reservoir flow field (Figure 1).^16^ Meanwhile, CO_2_ can be stored in the coal seam, greatly reducing the greenhouse effect. This technology has attracted much attention due to its safety, economy, environmental friendliness, and significant improvements in the rate of recovery of gas.

**Figure 1:**
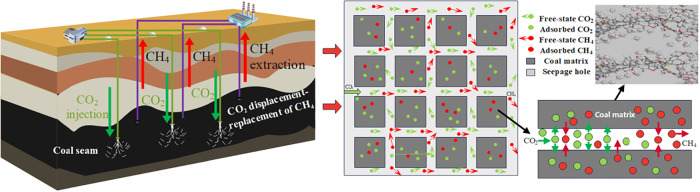
Schematic illustration of CO_2_-ECBM recovery mechanism via CO_2_ displacement–replacement.

Focusing on underground gas control in coal mines, Hu et al. proposed a technique for fracturing coal and rock and displacing coal-seam CH_4_ using liquid CO_2_ (Figure 2). They designed field experiments for displacing coal-seam CH_4_ by injecting CO_2_, which effectively shortened the predrainage time and reduced the drilling workload for drainage boreholes.^17–19^ The phase-transition process of liquid CO_2_ injected into the coal seam through boreholes is shown in Figure 3. In Figure 3A,B is the liquid-phase pressurization, B–C is the fluid–solid heat transfer, C–D is the liquid-phase seepage, D-E is the two-phase seepage and diffusion, and E-F is the gas-phase adsorption and diffusion. The effectiveness of liquid CO_2_ in enhancing the drainage of coal-seam CH_4_ mainly depends on the gas-phase adsorption-diffusion-seepage stage (D-F in Figure 3). This stage involves complex physical processes, including gas-phase seepage, diffusion, and adsorption of CO_2_, and features multifield coupling among the temperature field, pressure field, and gas concentration field.

**Figure 2:**
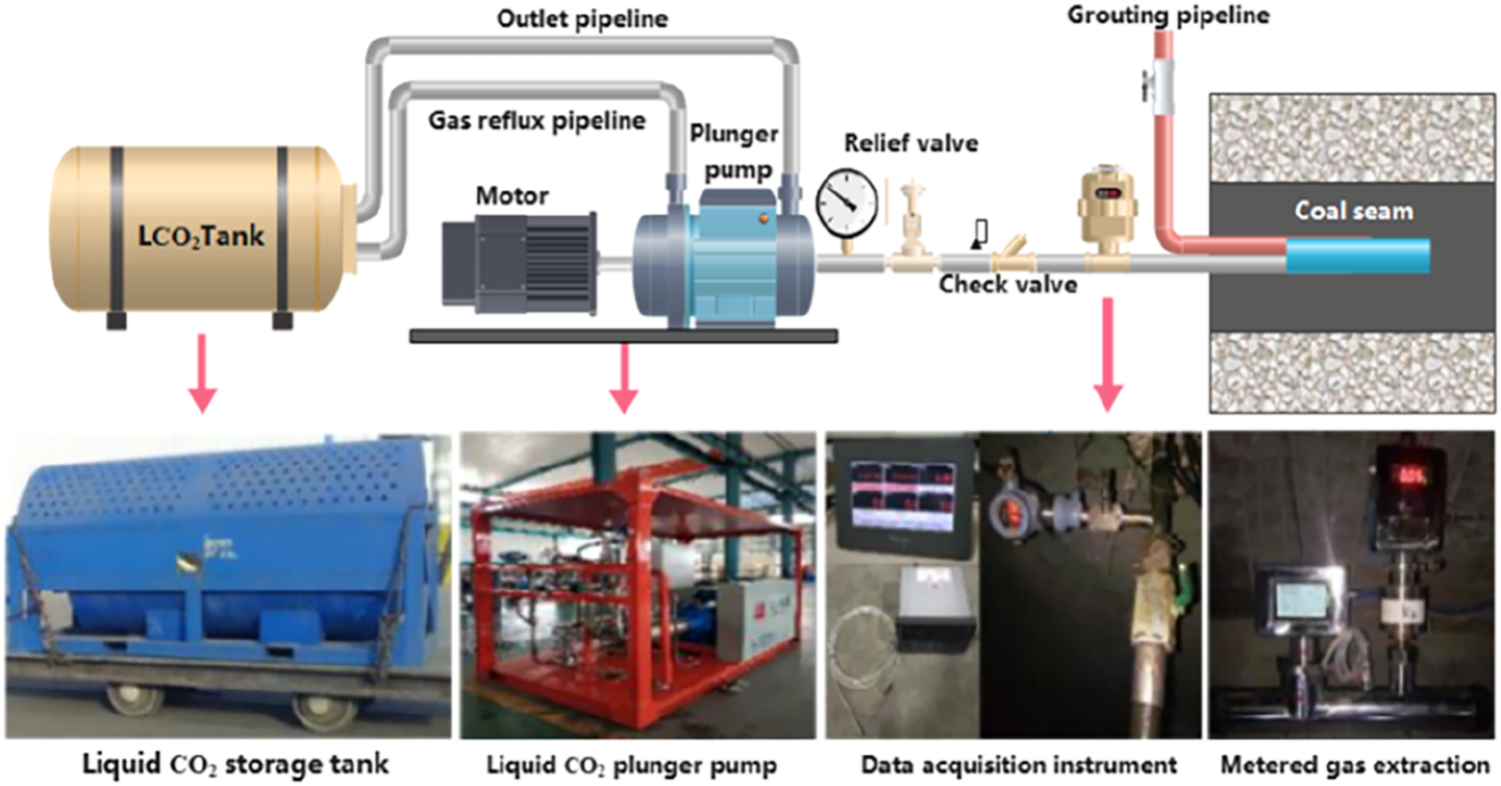
Technological process and equipment for liquid CO_2_-based displacement of CH_4_ in coal seams.

**Figure 3:**
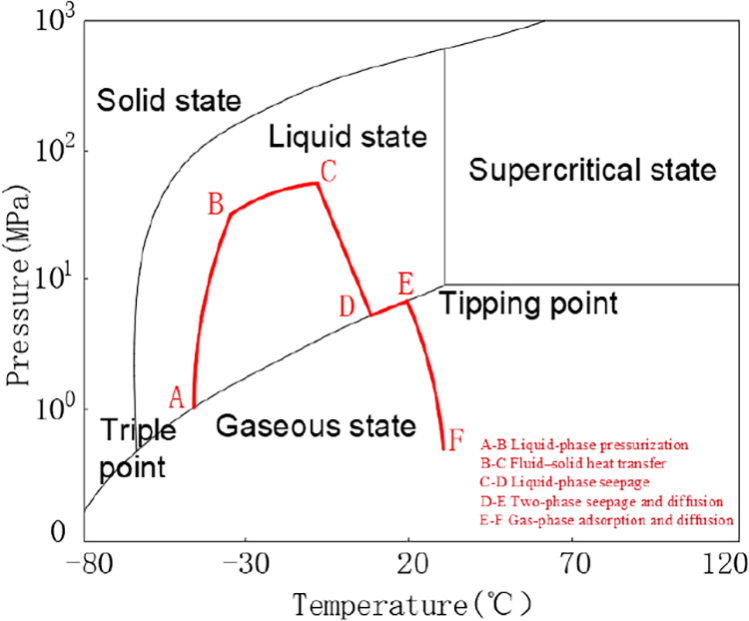
Phase change curve during the liquid CO_2_ pressure injection process.

The process of injecting CO_2_ to enhance the drainage of CH_4_ from coal seams involves the adsorption and diffusion of a binary gas mixture in coal. Diffusion acts as a bridge linking the adsorption, desorption, and seepage of two gases, thereby affecting the production of coal-bed methane (CBM) and the injection of CO_2_. Numerous scholars have conducted extensive experiments in this area.^20–26^ The research shows that, under the same conditions and for the same coal sample, the effective diffusion coefficient of CO_2_ is higher than that of CH_4_, whereas the diffusion of CH_4_ and CO_2_ within coals of different ranks is mainly controlled by surface diffusion in micropores.^27^ Under the same injection pressure, the diffusion coefficients of CO_2_, N_2_, and CH_4_ are found in the descending order of: CO_2_ > N_2_ > CH_4_. Yang Hongmin et al. investigated the displacement effects of different gases under varying injection pressures. Injection pressure affects the diffusion of CH_4_ in coal, and a dual-porosity (dual-pore) diffusion model can describe the entire adsorption–desorption-diffusion process of gas in coal particles more accurately than a single-porosity model.^28^ An Feng et al. studied the effect of the injection pressure of CO_2_ on the diffusion coefficient of gas, and concluded that higher pressure shortened the time to reach the maximum emissions of CH_4_, accelerated the CO_2_ breakthrough, increased the displacement efficiency, and improved the displacement performance.^29^ However, in experimental studies on the CO_2_-enhanced drainage of CH_4_ from coal seams, theoretical research on the diffusion behavior of the two gases remains limited and has mainly focused on factors such as injection pressure and type of gas. Moreover, diffusion behavior under different injection concentrations has yet to be fully clarified. Therefore, the present paper investigates the diffusion behavior of both gases during CO_2_-enhanced drainage of coal seam CH_4_, and it elucidates the spatiotemporal evolution of the adsorption of CO_2_ and the desorption of CH_4_. Moreover, the study analyzes the reciprocal evolutionary processes of CO_2_’s “diffusion–adsorption” and CH_4_’s “desorption-diffusion,” and develops a diffusion model for CO_2_-enhanced drainage of coal seam CH_4_. Furthermore, during the process, the diffusion behaviors of both gases are also characterized. The findings provide a theoretical basis for determining and optimizing key technical parameters for displacing coal seam CH_4_ by injecting CO_2_ and are of significant value for preventing hazards around mine gas and developing the resource of CBM.

## 2 Establishment of the Diffusion Model

Diffusion is a concentration equalization process in which the free motion of molecules causes substances to migrate from regions of high concentration to regions of low concentration.^30^ Diffusion’s behavior follows Fick’s first law of diffusion, as given by eq 1.

1 $$J=-D\frac{\partial c}{\partial x}$$

where *J* is the gas flux (g/(cm^2^·s)), *D* is the diffusion coefficient of gas in coal (cm^2^/s), *c* is the gas content in coal (g/cm^3^), $\frac{\partial c}{\partial x}$ is the gas concentration gradient along the diffusion direction ((g/m^3^)/m), and the minus sign indicates that diffusion occurs from high concentration region to low concentration region.

By applying Fick’s first law to a three-dimensional, unsteady flow field and invoking mass conservation and continuity, the second law of diffusion by Fick can be written as eq 2.

2 $$\frac{\partial c}{\partial t}=D\left(\frac{\partial^{2}c}{\partial x^{2}}+\frac{\partial^{2}c}{\partial y^{2}}+\frac{\partial^{2}c}{\partial z^{2}}\right)$$

By analyzing the NMR *T*_2_ spectra, one can directly or indirectly obtain the time evolution of the amounts of adsorbed- and free-phase CH_4_ and CO_2_ in the coal sample during displacement. Moreover, the amounts of substances (moles) of adsorbed and free CH_4_ and CO_2_ can then be calculated. According to Avogadro’s law, at the same temperature and pressure, the volumetric ratio of gases equals their molar ratio ( $\frac{V_{1}}{V_{2}}=\frac{n_{1}}{n_{2}}$ ). Accordingly, the percentages (or concentrations) of free-phase CH_4_ and free-phase CO_2_ in the total free gas within the coal sample can be determined.

The CO_2_ concentration at time *t* is given by eq 3.

3 $$C_{CO_{2}}^{t}=\frac{m_{CO_{2}}^{t}}{V_{CO_{2}}^{t}+V_{CH_{4}}^{t}}$$

The CH_4_ concentration at time *t* is given by eq 4.

4 $$C_{CH_{4}}^{t}=\frac{m_{CH_{4}}^{t}}{V_{CO_{2}}^{t}+V_{CH_{4}}^{t}}$$

Due to the heterogeneous nature of the actual pore-size distribution in coal samples, the average pore diameter *h* is assumed for the sake of simplifying the computational model and minimizing errors as much as possible. Then, the concentration gradient is given by $\frac{C}{h}$ . According to Fick’s law, the diffusion coefficient can be obtained by using eq 5.

5 $$D=\frac{h}{\mathrm{AC}}\cdot \frac{dQ}{dt}$$

where *A* is the cross-sectional area of the pore, *Q* is the amount of diffused CH_4_, and *t* is the diffusion time. Once the concentration gradient is known, eq 5 can be integrated to obtain the effective diffusion coefficient. To determine $\frac{dQ}{dt}$ , one can plot the amount of diffused gas against time, fit the curve, and then differentiate. The amount of diffused CH_4_ is the decrease in free-phase CH_4_, whereas the amount of diffused CO_2_ is the amount of CO_2_ entering the coal matrix.

## 3 Experimental Platform and Procedure

The experimental setup integrated a MacroMR12–150H–I large-bore nuclear magnetic resonance (NMR) imaging analyzer with a high-pressure gas-injection displacement platform. The main components of the experimental setup included an NMR imaging analyzer, a coal sample vessel, pneumatic valves, a gas storage tank, a pressure control unit, temperature sensors, a vacuum pump, and gas cylinders (Figure 4).

**Figure 4:**
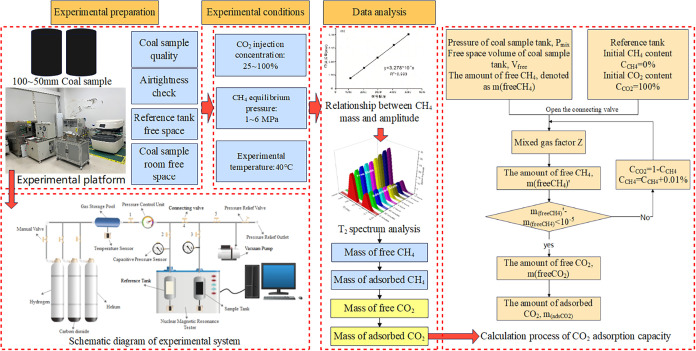
Schematic of the technical route for the calibration experiment of the coal body’s adsorption of CH_4_.

The coal samples selected for the experiments included long-flame coal, coking coal, and anthracite. To investigate the diffusion behavior during the displacement of CH_4_ by CO_2_, low-field NMR was used to perform *T*_2_ spectrum experiments on the three coal cores. Using the porosity calibration curve, we determined the porosity and pore-size distribution of the samples. In the NMR core analysis software, an empirical *T*_2_ cutoff was selected and entered to compute the saturation of bound fluid, saturation of free fluid, and permeability. The corresponding experimental results are presented in Table 1.

**Table 1:** Experimental Parameters and Corresponding Results for the Coal Cores

testing parameters	long-flame coal	coking coal	anthracite
Porosity/%	6.38	6.07	5.76
*T*_2_ cutoff value/ms	1.103	0.82	0.691
Bound flow saturation/%	55.7079	50.98	56.093
Free fluid saturation/%	44.4018	49.02	43.906
Permeability/mD	0.3677	0.269	0.0673

Next, NMR experiments for the calibration gas of CH_4_ were conducted within the pressure ranges of 1–6 MPa. The prepared 100 mm × 50 mm coal samples were subjected to the coal’s quality analysis and measurements of true density and apparent density. The mass of the loaded coal sample, the free volume of the sample chamber, *V*_1_ = 33.39 mL, and the free volume of the reference tank, *V*_2_ = 44.87 mL, were recorded.

Finally, experiments were conducted to assess the effect of the injection concentration of CO_2_ on the diffusion of CH_4_ during its displacement. The three selected coal cores were dried in an oven at 80 °C. After drying, experiments for displacing coal-seam CH_4_ by gaseous CO_2_ were performed. The experimental temperature was set to be 40 °C. To avoid pressure-difference effects, an injection pressure of 2 MPa, which was equal to the equilibrium pressure for the adsorption of methane (2 MPa) in coal cores, was used to inject pure CO_2_, a CO_2_/He mixture of 25%/75%, a CO_2_/He mixture of 50%/50%, and a CO_2_/He mixture of 75%/25% so that the CO_2_-enhanced extraction of CH_4_ from coal could be conducted.

## 4 NMR CH_4_ Calibration Experiment

Prior to the experiment, the lines were evacuated for 6 h, after which methane was injected into the sample chamber at a pressure of 1–6 MPa to acquire NMR signals under different conditions for pressure and temperature. Taking 40 °C as an example, the *T*_2_ spectrum of free methane is shown in Figure 5.

**Figure 5:**
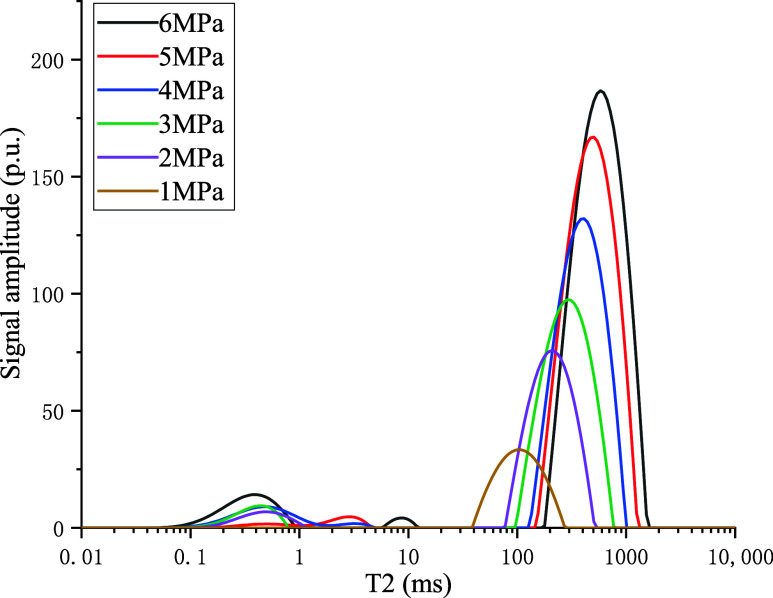
*T*_2_ curve of free state CH_4_ at 40 °C.

The NMR experiments of free gas indicated that the *T*_2_ spectrum of free gas exhibited only one characteristic peak, whereas both the peak area (integral amplitude) and the transverse relaxation time (*T*_2_) were proportional to gas pressure. This meant that when the pressure increased, the peak shifted to longer *T*_2_ (rightward). Therefore, in the NMR spectra of coal-gas adsorption/desorption, one can distinguish the spectral ranges corresponding to adsorbed and free gases.

Based on the *T*_2_ spectra of free CH_4_, the signal amplitude integration corresponding to free CH_4_ at 1- 6 MPa was extracted to plot a fitted curve of pressure versus *T*_2_ signal amplitude integrations of free CH_4_, as shown in Figure 6.

**Figure 6:**
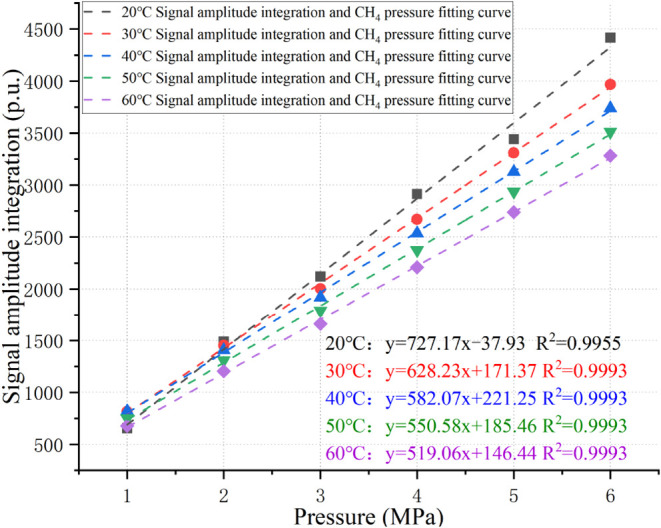
Relationship between pressure and signal amplitude integration.

According to the natural gas calculation of the compression factor, the compression factor *Z* was calculated under different pressures, and the corresponding results are listed in Table 2. Then, the mass of CH_4_ under different pressure and temperature conditions was calculated by using the real-gas equation of state PV = *ZnRT*. Therefore, by this method, the measured relationship between pressure and the *T*_2_ signal amplitude integration of free CH_4_ can be converted into a correspondence between CH_4_ mass and the *T*_2_ signal amplitude integration of free CH_4_, as shown in Figure 7.

**Figure 7:**
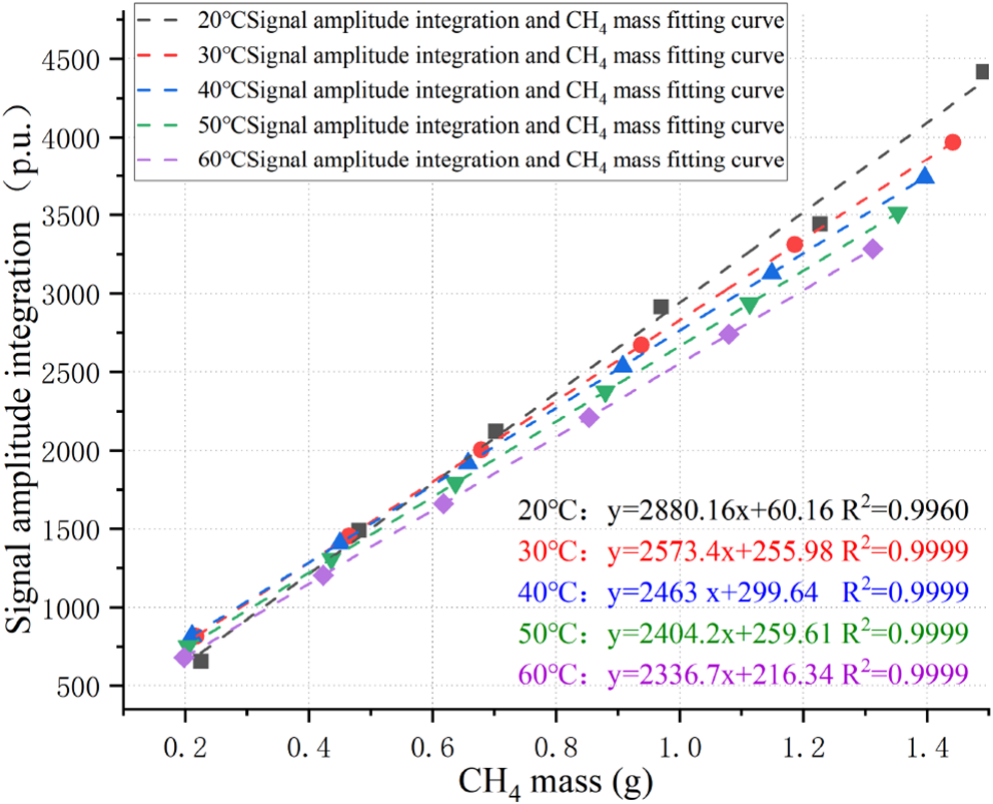
Integral fitting curve of free-state CH_4_ mass and signal amplitude integration.

**Table 2:** Relationship between Compressibility Factor *Z* and Methane Mass

pressure/MPa	*Z*	CH_4_ mass/g	amplitude integral
0.99	0.98	0.2254	656.6120
2.07	0.96	0.4811	1490.8610
2.96	0.94	0.7025	2121.6470
4	0.92	0.9700	2914.1360
4.95	0.9	1.2271	3442.1290
5.95	0.89	1.4915	4417.5120
6.94	0.87	1.7797	5047.4240
7.96	0.86	2.0650	5822.2680

The results reveal that at different temperatures, the CH_4_ mass is proportional to the integrated signal amplitude of the NMR *T*_2_ spectrum. In other words, the greater the mass of free CH_4_ is, the larger the integrated signal amplitude. The relationship is linear, with coefficients of determination of *R* ^2^ all exceeding 0.9960. Therefore, by using the fitted curve between the integrated *T*_2_ signal amplitude and CH_4_ mass, the CH_4_ mass under different pressure conditions can be calculated, thereby completing the calibration of free-state CH_4_.

## 5 Experimental Study on the Effect of Injection Concentration of CO_2_ on the Diffusion of CH_4_

The experimental temperature was set to be 40 °C. To avoid pressure-difference effects, an injection pressure of 2 MPa, which was equal to the equilibrium pressure for the adsorption of methane (2 MPa) in coal cores, was used to inject pure CO_2_, a CO_2_/He mixture of 25%/75%, a CO_2_/He mixture of 50%/50%, and a CO_2_/He mixture of 75%/25%. To calculate the amount of CH_4_ desorbed during the CO_2_ gas displacement–replacement of CH_4_, NMR T_2_ spectra were first acquired under each set of conditions. After obtaining the *T*_2_ spectra, the relationship between the amplitude of the integrated *T*_2_ signal and the free-phase CH_4_ was used to determine the time evolution of the desorption of CH_4_. Meanwhile, residual adsorbed CH_4_, the amount of adsorbed CO_2_, and the amount of free-phase CO_2_ could be indirectly calculated.

### 5.1 Analysis of the Amount of Desorbed CH_4_

#### 5.1.1 Analysis of the Stage Desorption Amount of CH_4_

The stage desorption amounts of CH_4_ in coal samples under various injection concentrations of CO_2_ are shown in Figure 8.

**Figure 8:**
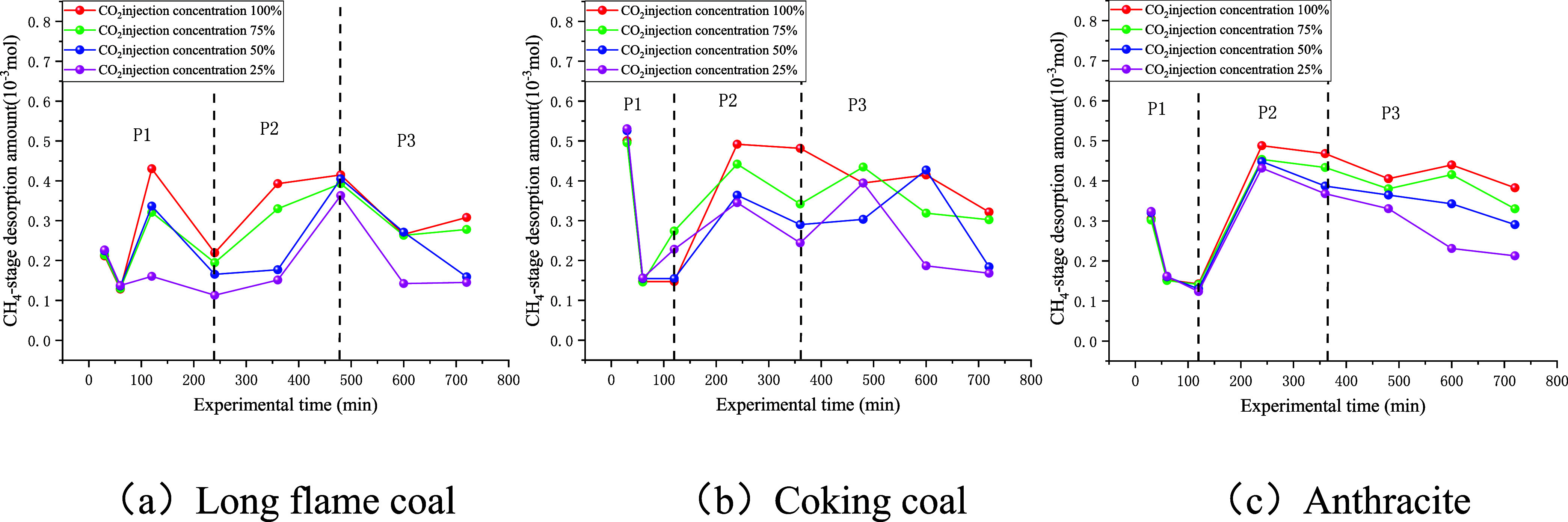
Stage desorption amount curve of CH_4_.

As shown in Figure 8, during Stage P_1_, the stage desorption amount of CH_4_ decreased with an increase in experimental time. During Stage P_2_, the amount of desorbed methane increased steadily with time. Upon entering Stage P_3_, the amount of desorbed methane gradually decreased with time. This is because, in P_1_, when the connecting valve was opened and CO_2_ entered the coal sample tank, the partial pressure of CH_4_ dropped, which was due to the immediate displacement of the free-phase CH_4_. Then, the replacement of adsorbed CH_4_ began. By this time, most of the free CH_4_ had already been expelled, resulting in a large initial-stage desorption amount that declined rapidly. Therefore, this stage reflected both the displacement and the replacement effects of CO_2_ on CH_4_. When P_2_ was reached, free-phase CH_4_ in coal continued to decrease, whereas replacement of CO_2_ became dominant. Although the stage desorption amount of CH_4_ still increased, it tended to stabilize. By P_3_, the replacement of CO_2_ was essentially complete, and the amounts of both free and adsorbed CH_4_ remaining in the coal sample became small. The replacement effect continued to weaken, leading to a continual decrease in the stage desorption amount of CH_4_.

In addition, the duration of P_1_ for long-flame coal was greater than that for coking coal and anthracite, whereas the duration of its P_2_ stage was noticeably shorter. This can be attributed to differences in permeabilities. The results showed that the long-flame coal core had the highest permeability among the three coal samples, providing more flow pathways for CO_2_ and CH_4_, and making its P_1_ stage significantly longer than those of the other two coals. Moreover, with the increase in the injection concentration of CO_2_, the stage desorption amount of CH_4_ increased for all three coals. However, at 100 and 75% CO_2_, the curves exhibited crossover behavior, and the incremental increase in stage desorption of CH_4_ became small. This is because the injection of CO_2_ gas competes with CH_4_ gas for adsorption sites on the surface and pores of the coal samples. As the concentration of CO_2_ increases, the adsorption amount of CO_2_ in the coal sample increases, thereby driving the desorption of CH_4_. After reaching a concentration of 75%, the CO_2_ concentration in the system is sufficient to effectively displace CH_4_, and further increasing the CO_2_ concentration has a limited promoting effect on CH_4_ desorption.

#### 5.1.2 Analysis of the Cumulative Desorption Amount of CH_4_

The cumulative desorption of CH_4_ in coal samples under different injection concentrations of CO_2_ is shown in Figure 9. The experimental data at 600 min were selected as an example to compile Table 3.

**Figure 9:**
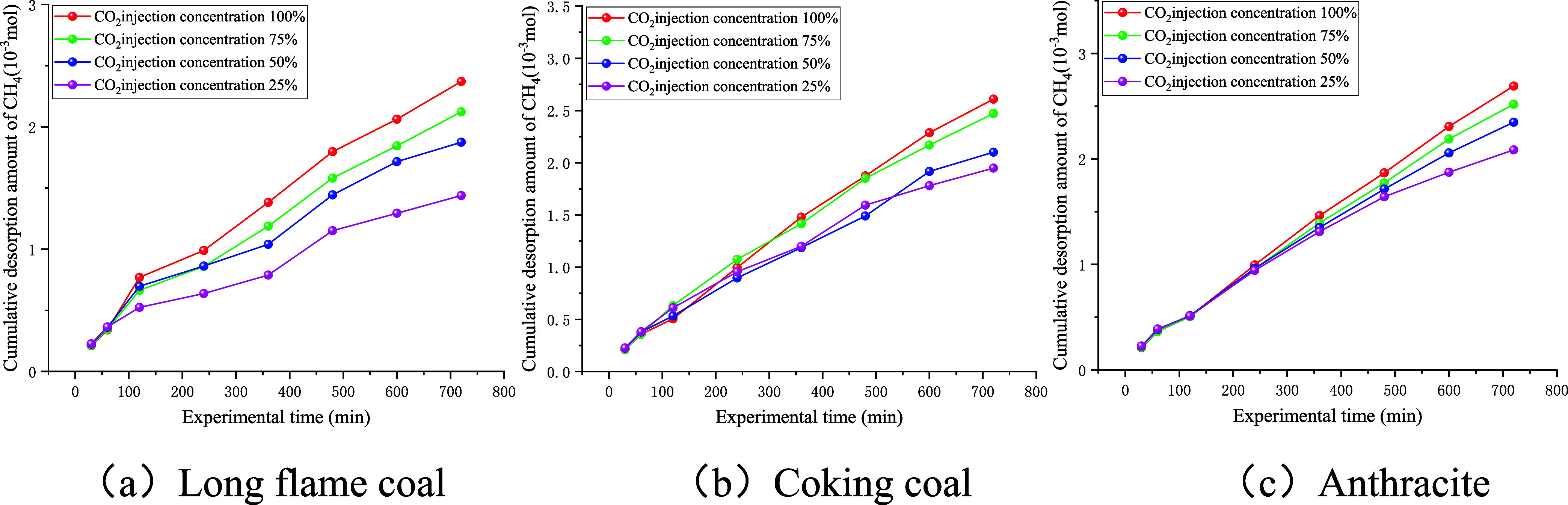
Cumulative desorption amount curve of CH_4_

**Table 3:** Influence of CO_2_’s Injection Concentration on the Cumulative Desorbed Amount of CH_4_

	cumulative desorbed amount of CH_4_(10^–3^ mol)		
CO_2_ injection concentrations	long-flame coal	coking coal	anthracite
100%	2.06	2.65	2.30
75%	1.83	2.38	2.18
50%	1.67	2.21	2.05
25%	0.94	1.37	1.87

The cumulative desorption amount of CH_4_ for all three coal samples increased with the experimental time. Moreover, as the injection concentration of CO_2_ decreased, the cumulative desorption amount of CH_4_ also decreased. These differences arose because the surface characteristics of the adsorbent have a significant effect on the adsorption behavior of two gases. CO_2_ molecules have strong polarity characteristics and are more easily adsorbed, while CH_4_ is a nonpolar molecule with weaker adsorption ability on polar surfaces. In addition, the adsorption heat of CO_2_ is usually higher than that of CH_4_. This means that CO_2_ tends to form a stable adsorption state on the adsorbent surface, while the lower adsorption heat of CH_4_ puts it at a disadvantage in competition. When both exist simultaneously, CO_2_ can more effectively occupy adsorption sites, thereby inhibiting the adsorption of CH_4_. When the injection concentration of CO_2_ was reduced from 100 to 25%, the cumulative CH_4_ desorption dropped by 54.10% for long-flame coal, 48.01% for coking coal, and 18.83% for anthracite. This indicated that cumulative CH_4_ desorption was influenced not only by the injection concentration of CO_2_ but also by the degree of coalification.

### 5.2 Analysis of the Adsorbed Amount of CO_2_

#### 5.2.1 Analysis of the Stage Adsorption Amount of CO_2_

As shown in Figure 10, the stage adsorption amount of CO_2_ in coal samples increased rapidly with experimental time and, at a later stage, tended to stabilize. Moreover, as the injection concentration of CO_2_ increased, the stage adsorption amount of CO_2_ also increased.

**Figure 10:**
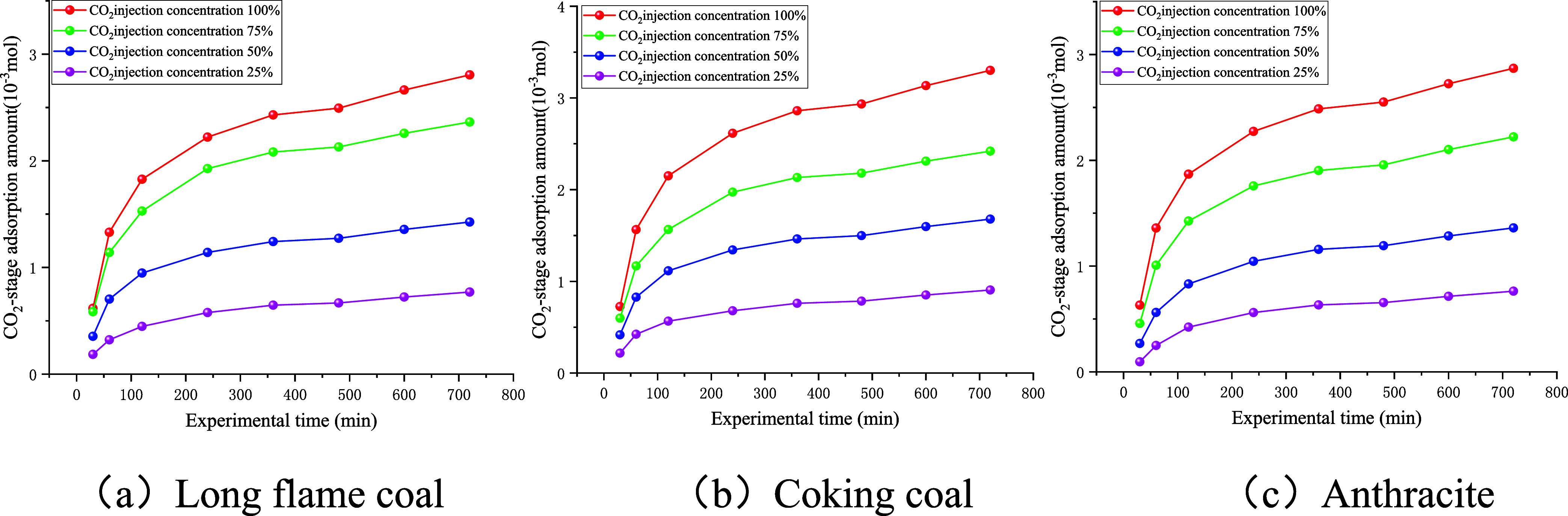
Stage adsorption amount curve of CO_2_

#### 5.2.2 Analysis of the Cumulative Adsorbed Amount of CO_2_

As shown in Figure 11, the cumulative CO_2_ adsorption in coal samples was directly proportional to the experimental time and increased with higher injection concentration of CO_2_. The experimental data at 600 min were selected to compile Table 4.

**Figure 11:**
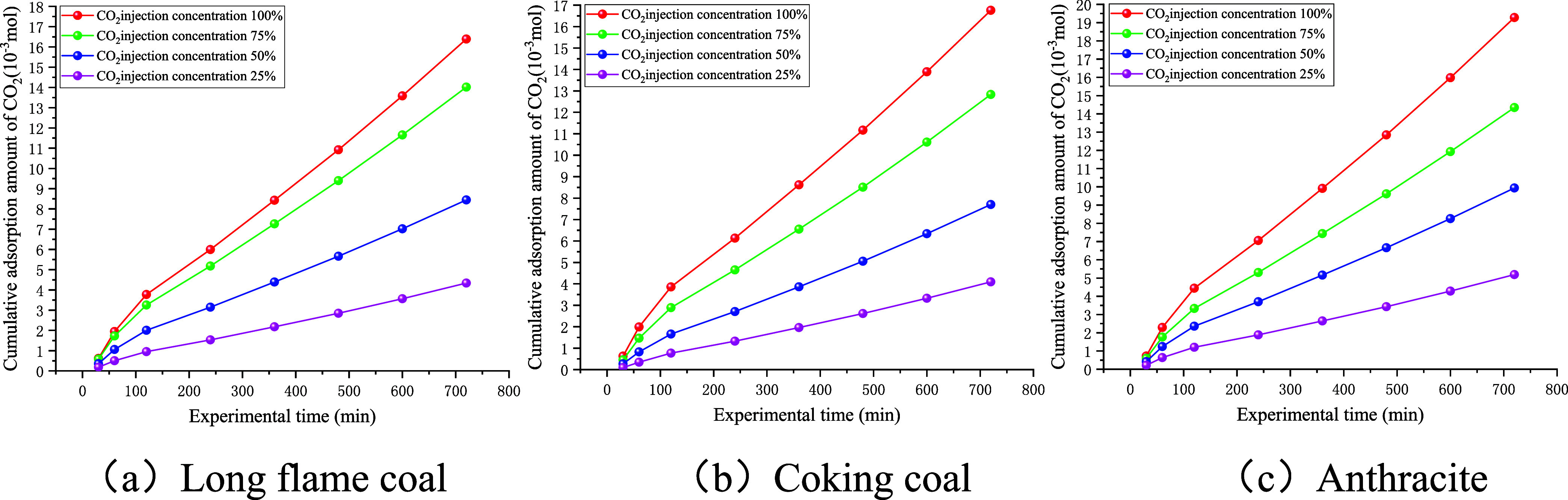
Cumulative desorption amount curve of CO_2_

**Table 4:** Influence of CO_2_’s Injection Concentration on the Cumulative Adsorbed Amount of CO_2_

	cumulative adsorbed amount of CO_2_(10^–3^ mol)		
CO_2_ injection concentrations	long-flame coal	coking coal	anthracite
100%	13.58	13.89	15.98
75%	11.65	10.61	11.92
50%	7.02	6.34	8.25
25%	3.56	3.32	4.28

As shown by the data presented in Table 4, the cumulative adsorbed amount of CO_2_ for all three coal samples decreased with the decrease in the injection concentration of CO_2_. When the CO_2_ injection concentration was reduced from 100 to 25%, the partial pressure of CO_2_ dropped by 75%. Under these conditions, the cumulative adsorbed amount of CO_2_ decreased by 73.73% for long-flame coal, 76.03% for coking coal, and 73.20% for anthracite. Therefore, the decline in the cumulative adsorbed amount of CO_2_ was approximately proportional to the decrease in the partial pressure of CO_2_. For example, a 25% reduction in the CO_2_ partial pressure led to about a 25% reduction in the cumulative adsorbed amount of CO_2_, whereas a 50% reduction in the partial pressure of CO_2_ led to about a 50% reduction in the cumulative adsorbed amount of CO_2_. This indicates that the CO_2_ partial pressure has a strong influence on the cumulative adsorbed amount of CO_2_.

### 5.3 Comparative Analysis of CO_2_ Adsorption and Residual CH_4_ Adsorption

As shown in Figure 12, the adsorption amounts of both gases were directly proportional to the injection concentration of CO_2_. The slope of the CO_2_ adsorption curve was markedly higher than that of the residual CH_4_ adsorption curve, indicating that the injection concentration of CO_2_ had a relatively small effect on residual CH_4_ adsorption (but a much larger effect on the adsorption of CO_2_). Moreover, during displacement, the coal adsorbed far more CO_2_ than CH_4_. To more clearly illustrate the difference between the adsorbed amounts of the two gases, data are presented in Table 5.

**Figure 12:**
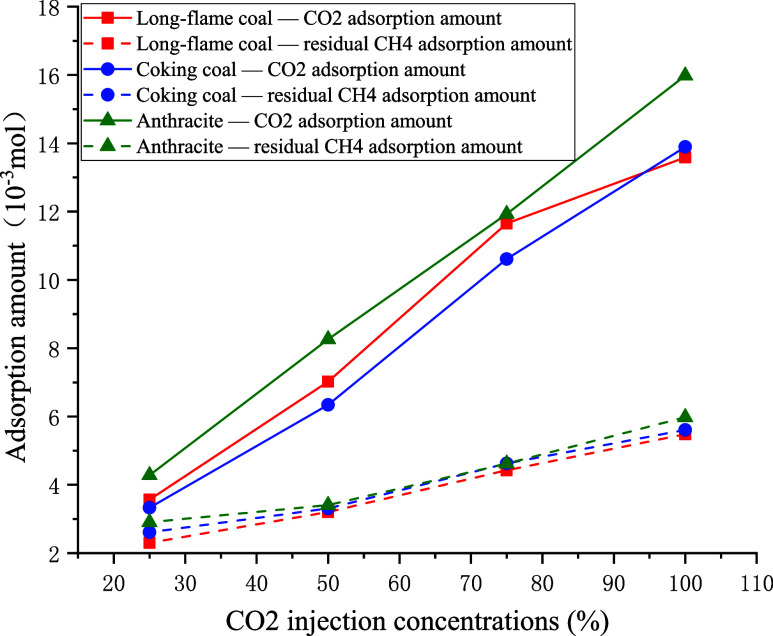
Relationship between CO_2_’s injection concentration and the adsorption of CO_2_ and CH_4_.

**Table 5:** Relationship between the Injection Concentration of CO_2_ and the Difference between the Adsorbed Amounts of CO_2_ and CH_4_

	difference between CO_2_ and CH_4_ adsorption amounts (10^–3^ mol)		
CO_2_ injection concentrations	long-flame coal	coking coal	anthracite
100%	8.11	8.28	10.00
75%	7.22	5.98	7.30
50%	3.81	3.03	4.85
25%	1.26	0.71	1.37

As shown by the data presented in Table 5, for all three coal samples, the difference between the amounts of the two gases adsorbed decreased with the decrease in the injection concentration of CO_2_, indicating that a lower injection concentration of CO_2_ led to poorer displacement performance. For 100% CO_2_, this difference reached its maximum for all three coals (the adsorbed amounts were 8.11 × 10^–3^ mol for long-flame coal, 8.28 × 10^–3^ mol for coking coal, and 10.00 × 10^–3^ mol for anthracite).

### 5.4 Results and Discussion of Diffusion Coefficients

#### 5.4.1 Diffusion Coefficient of Methane

As shown in Figure 13, the amount of diffused methane increased with the experimental time and displayed an upward trend with the increase in the injection concentration of CO_2_. As shown by the data presented in Table 6, the fitted relationships between the amount of CH_4_ diffused and experimental time for the three coal samples all conformed to the logarithmic correlation of: *Q* = *a* · ln­(*t*) - *b*, with good correlation that met the requirements for experimental accuracy. By differentiating the fitted functions and substituting into eqs 5,6 can be obtained, which can be used to calculate the diffusion coefficient of CH_4_. The calculation results are shown in Figure 14.

6 $$D_{CH_{4}}=a\cdot \frac{h}{A\cdot C\cdot t}$$

Where DC_CH_4__ is the diffusion coefficient of CH_4_, and *a* is the coefficient of the fitting function.

**Figure 13:**
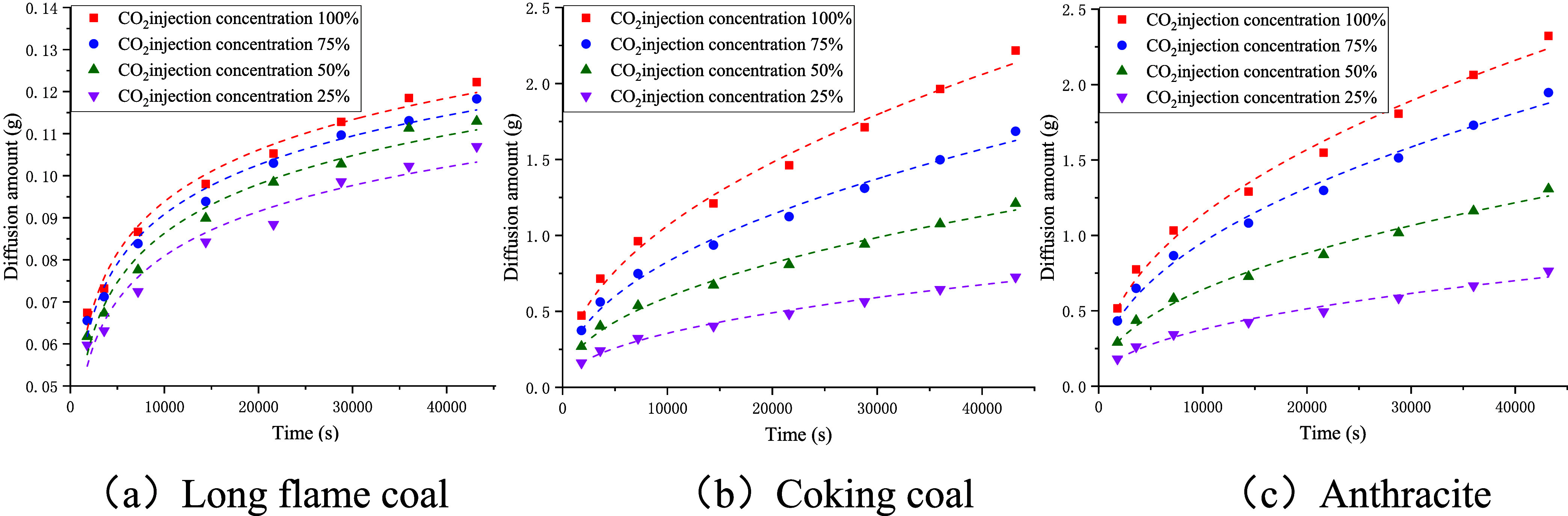
Fitted relationship between the amount of diffused CH_4_ and the experimental time.

**Figure 14:**
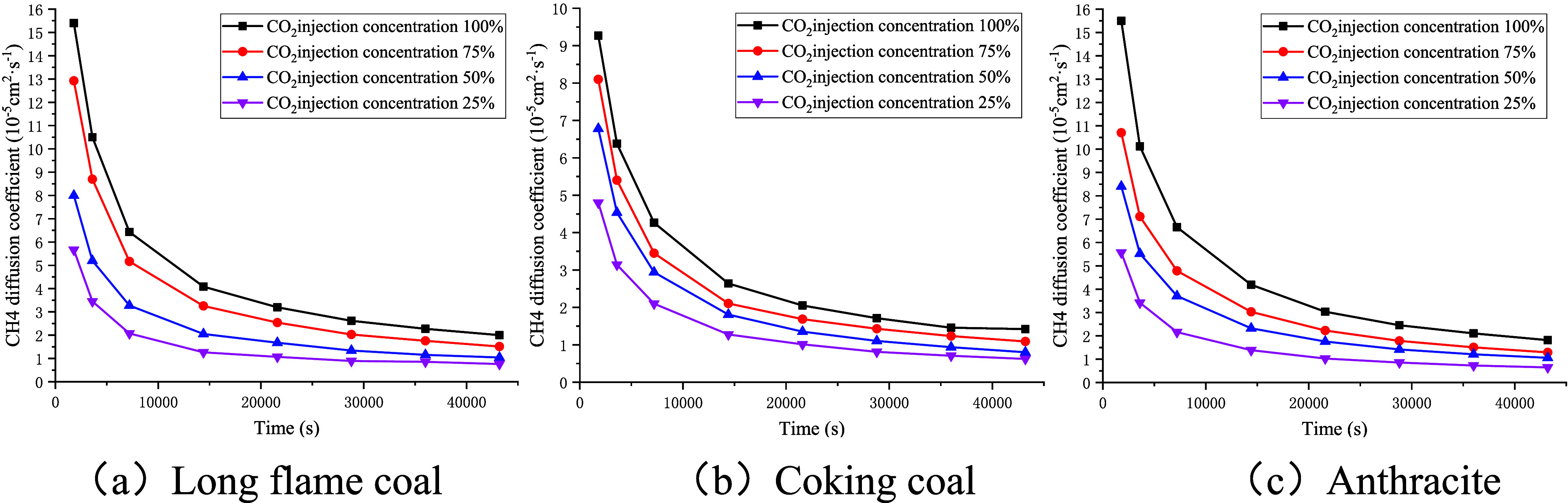
Relationship curve between CH_4_’s diffusion coefficient and experimental time.

**Table 6:** Fitted Relationship between the Amount of Diffused CH_4_ and the Experimental Time

	long-flame coal			coking coal			anthracite		
CO_2_ injection concentrations	*a*	*b*	*R* ^2^	*a*	*b*	*R* ^2^	*a*	*b*	*R* ^2^
100%	0.018	0.069	0.985	0.016	0.064	0.993	0.014	0.020	0.994
75%	0.017	0.065	0.985	0.016	0.066	0.982	0.016	0.056	0.994
50%	0.017	0.069	0.979	0.015	0.062	0.994	0.016	0.059	0.994
25%	0.015	0.060	0.963	0.015	0.061	0.995	0.015	0.056	0.994

As shown in Figure 14, the diffusion coefficient of methane showed a rapid decline with the increase in experimental time and tended to level off. The higher the injection concentration of CO_2_, the larger the diffusion coefficient of methane, while the volume fraction of free-phase CH_4_ decreased with the increase in the injection concentration of CO_2_. Using the data at an experimental time of 36,000 s as an example, when the injection concentration of CO_2_ decreased from 100 to 25%, the diffusion coefficient of methane for long-flame coal dropped from 2.27 × 10^–5^ to 0.86 × 10^–5^ cm^2^/s, whereas the volume fraction of free-phase CH_4_ increased from 14.75 to 36.09%. For coking coal, the diffusion coefficient of methane decreased from 1.46 × 10^–5^ to 0.71 × 10^–5^ cm^2^/s, with the volume fraction of free-phase CH_4_ increasing from 20.15 to 38.52%. Meanwhile, for anthracite, the diffusion coefficient of methane decreased from 2.11 × 10^–5^ to 0.73 × 10^–5^ cm^2^/s, while the volume fraction of free-phase CH_4_ increased from 17.10 to 41.60%.

At the initial stage of injection, as CO_2_ continuously entered the coal sample, it rapidly displaced a large amount of free-phase CH_4_, leading to an increase in the partial pressure of CH_4_ within the pores. With the increase in partial pressure, the mean free path of CH_4_ molecules decreased, their density increased, and the concentration gradient of CH_4_ within the coal matrix became larger, enhancing its diffusion capacity. In addition, the heat released by the adsorption of CO_2_ promoted the desorption of adsorbed CH_4_, increasing the amount of free-phase CH_4_. Furthermore, the larger concentration gradient of methane was favorable for diffusion, resulting in a relatively high diffusion coefficient at the beginning of the experiment.

In the midinjection stage, the replacement effect of CO_2_ expelled a large amount of free-phase CH_4_ from the pores. Moreover, CO_2_ molecules competed with CH_4_ in the micropores for adsorption sites, replacing CH_4_. Meanwhile, the heat released by the adsorption of CO_2_ activated the CH_4_ molecules, facilitating their desorption into the free phase and their subsequent removal with the gas flow. The partial pressure of CO_2_ within the coal sample increased rapidly, and because the molecular (kinetic) diameter of CO_2_ was smaller than that of CH_4_, CO_2_ entered the micropores more readily than did methane. At the same time, as CO_2_ continued to diffuse–adsorb and CH_4_ continued to desorb-diffuse, the concentration of CH_4_ in the coal sample gradually decreased, while the concentration gradient of CH_4_ diminished. Furthermore, diffusion weakened and the diffusion coefficient of methane decreased continuously.

In the later stage of gas injection, a large amount of adsorbed CH_4_ was replaced and converted into free gas, which was then released from the coal body. The content of CH_4_ within the coal body decreased sharply, while the adsorption of CO_2_ gradually reached equilibrium. As the concentration gradient of CH_4_ within the coal body narrowed, the diffusion coefficient of CH_4_ stabilized in a later stage.

#### 5.4.2 Diffusion Coefficient of Carbon Dioxide

As shown in Figure 15, the amount of diffused CO_2_ increased with the experimental time and exhibited an upward trend with the increase in the injection concentration of CO_2_ gas. As presented in Table 7, the fitting results for the amount of diffused CO_2_ for the three coal samples conformed to a power function relationship, expressed as *Q* = *a*· *x^b^*, with a good correlation coefficient. By utilizing the fitting results, we can differentiate them and substitute them into eq 5 to obtain the diffusion coefficient equation for the CO_2_ gas (eq 7). The calculated results are listed in Figure 16.

7 $$D_{CO_{2}}=a\cdot b\cdot \frac{h\cdot t^{\left(b-1\right)}}{\mathrm{AC}}$$

where *D*_CO_2__ is the diffusion coefficient of CO_2_, and *a* and *b* are the coefficients of the fitting function.

**Figure 15:**
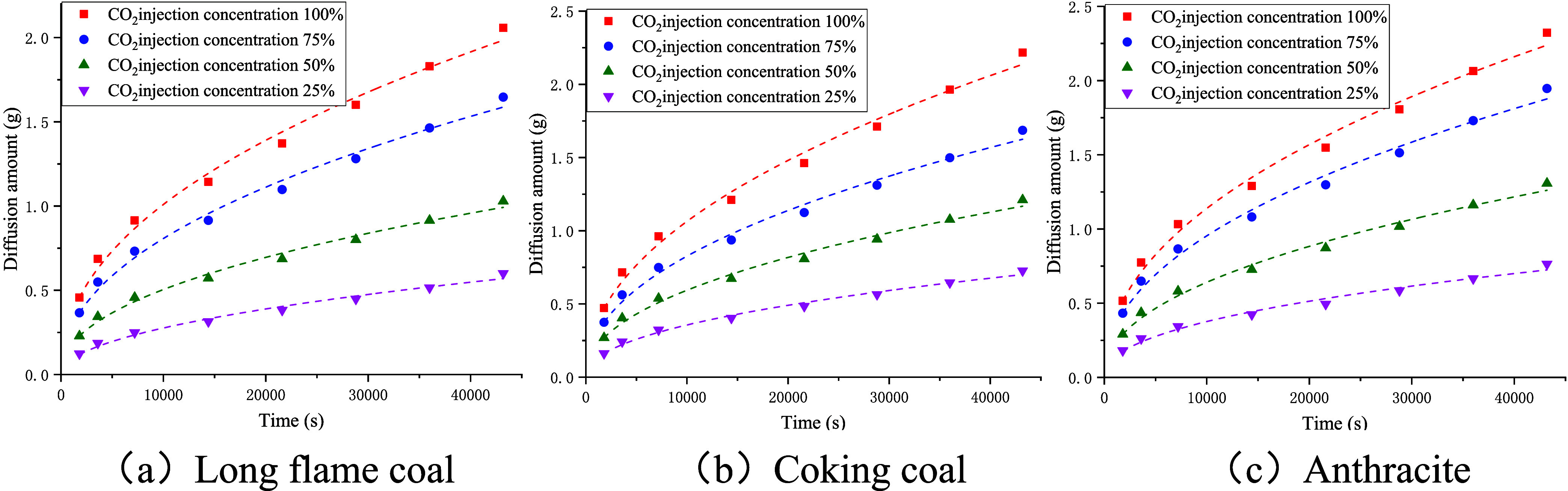
Fitted relationship between the amount of diffused CO_2_ and the experimental time.

**Figure 16:**
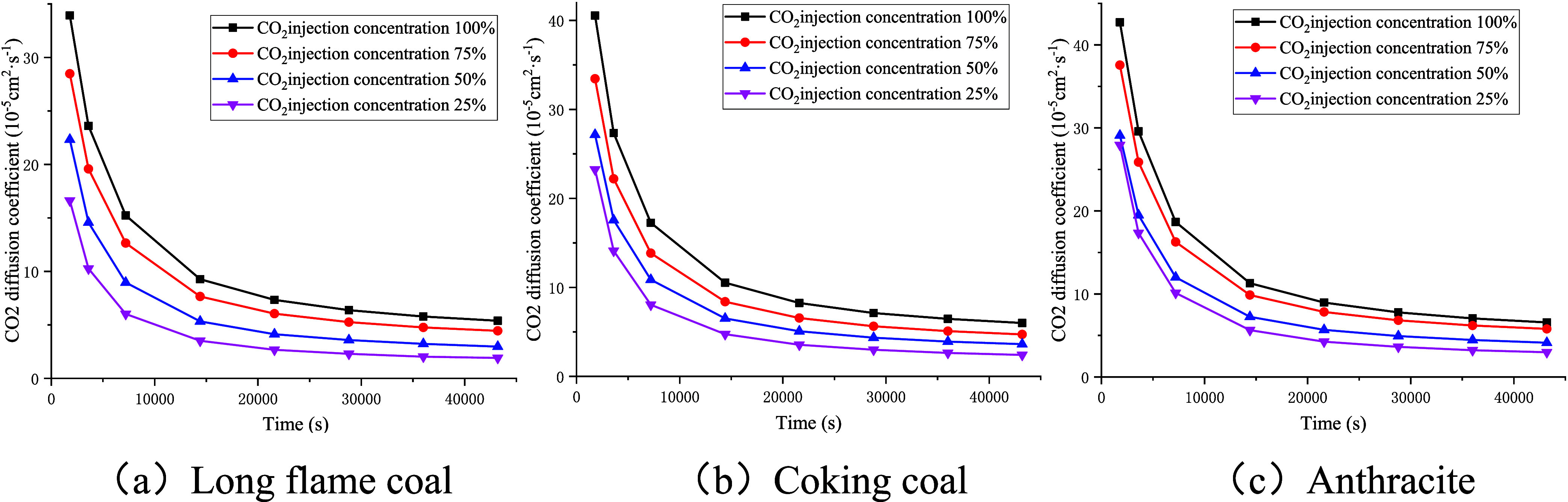
Relationship curve between CO_2_’s diffusion coefficient and experimental time.

**Table 7:** Fitted Relationship between the Amount of Diffused CO_2_ and the Experimental Time

	long-flame coal			coking coal			anthracite		
CO_2_ injection concentrations	*a*	*b*	*R* ^2^	*a*	*b*	*R* ^2^	*a*	*b*	*R* ^2^
100%	0.014	0.462	0.991	0.013	0.477	0.991	0.016	0.462	0.991
75%	0.011	0.462	0.991	0.012	0.462	0.991	0.014	0.462	0.991
50%	0.007	0.462	0.991	0.008	0.462	0.991	0.009	0.462	0.991
25%	0.003	0.494	0.986	0.005	0.462	0.991	0.006	0.447	0.984

As shown in Figure 16, the diffusion coefficient of CO_2_ gas exhibited a rapid decline, followed by a trend toward stabilization with the progression of the experiment. The diffusion coefficient of CO_2_ decreased with the increase in the injection concentration of CO_2_, while the volume fraction of free CO_2_ increased with higher injection concentrations of CO_2_. Taking the experimental data at 36,000 s as an example, it can be seen that when the injection concentration of CO_2_ decreased from 100% to 25%, the diffusion coefficient of CO_2_ for long-flame coal decreased from 5.78 × 10^–5^ to 2.03 × 10^–5^ cm^2^/s, whereas the volume fraction of free CO_2_ decreased from 89.57 to 71.81%. For coking coal, the diffusion coefficient of CO_2_ decreased from 6.46 × 10^–5^ to 2.64 × 10^–5^ cm^2^/s, while the volume fraction of free CO_2_ decreased from 89.06 to 69.27%. For anthracite, the diffusion coefficient of CO_2_ decreased from 7.78 × 10^–5^ to 3.63 × 10^–5^ cm^2^/s, with the volume fraction of free CO_2_ decreasing from 82.12 to 57.03%.

This is because, in the initial stage of gas injection, the injected CO_2_ gas creates a significant concentration gradient within the fractures, resulting in a larger diffusion coefficient for CO_2_. In the midstage of gas injection, since the diffusion coefficient of CO_2_ was much larger than that of CH_4_, the volume fraction of free CO_2_ entering the coal body continuously increased with the passage of experimental time, while the volume fraction of free CH_4_ decreased. In the later stage of gas injection, once the CO_2_ within the coal body reached adsorption saturation, the concentration gradient of CO_2_ gradually diminished with an increase in experimental time. This led to a gradual reduction in the diffusion coefficient of CO_2_, which stabilized over time.

## 6 Conclusions

This study utilized the controlled variable method to investigate the effect of the CO_2_ injection concentration on CH_4_ diffusion under constant temperature conditions, without considering the influences of in situ stress and pressure differences. Based on the analysis of the variation patterns of adsorbed CO_2_ and desorbed CH_4_ during the experiment, the diffusion behaviors of the two gases were studied, revealing the evolutionary processes of CO_2_ “diffusion-adsorption” and CH_4_ “desorption-diffusion.” Additionally, a gas diffusion model was established and validated by using CO_2_ displacement experiments for CH_4_ diffusion in coal seams. The main conclusions drawn are as follows.

According to the stage desorption amount curve of CH_4_, the process of injecting CO_2_ to enhance the drainage of CH_4_ from coal seams can be divided into three stages: a stage where replacement and displacement effects act jointly; a replacement-effect-dominated stage; and a steady stage. In addition, based on the fitted results of the diffusion quantities of the two gases over time, calculation models were established for the CH_4_ diffusion coefficient and the CO_2_ diffusion coefficient, represented as follows: $D_{{\mathrm{CH}}_{4}}=a\cdot \frac{h}{A\cdot C\cdot t}$ and $D_{{\mathrm{CO}}_{2}}=a\cdot b\cdot \frac{h\cdot t^{\left(b-1\right)}}{\mathrm{AC}}$ , respectively.

Using the established calculation models, an analysis was conducted on the diffusion behaviors of the two gases under different CO_2_ injection concentration conditions. It was found that the diffusion coefficients of both gases decreased rapidly with increasing experimental time and eventually stabilized. As the CO_2_ injection concentration increased, the diffusion coefficient of CH_4_ exhibited an upward trend, while the diffusion coefficient of CO_2_ showed a downward trend. This is due to the increase in the CO_2_ concentration, which enhances the intermolecular interactions and competitive adsorption in the gas mixture, while also causing changes in gas density.

This study elucidates the adsorption, desorption, and diffusion behaviors of CO_2_ and CH_4_ during the process of the extraction of CO_2_-enhanced methane from coal seams under different CO_2_ injection concentrations. It not only enhances the theoretical framework for gas migration during CO_2_ displacement and replacement of coal seam CH_4_ but also provides valuable guidance for the optimal selection of CO_2_ injection concentrations in field applications, contributing to improved efficiency in methane extraction from coal seams.

## References

[1] Wang S. (2020). On the status
of coal as a primary energy source in China and green mining. China Coal.

[2] Ge S., Liu S., Fan J., Zhao J., Teng T. (2024). Key technological system
for low-carbon modern coal-based energy development. J. China Coal Soc..

[3] Yuan L., Jiang Y., Wang K., Zhao X., Hao X., Xu C. (2018). Scientific
considerations for precise development and utilization
of resources in China’s closed/abandoned mines. J. China Coal Soc..

[4] Wang E., Zhang G., Zhang C., Li Z. (2022). Research progress and
prospects of theory and technology for prevention and control of coal
and gas outbursts in China. J. China Coal Soc..

[5] Wang F., Kobina F. (2025). The Influence of Geological
Factors and Transmission
Fluids on the Exploitation of Reservoir Geothermal Resources: Factor
Discussion and Mechanism Analysis. Reserv. Sci..

[6] Yang H., Feng C., Chen L. (2016). Analysis of displacement–driving
effects and their transformation mechanisms in nitrogen injection
simulation experiments in coal seams. J. China
Coal Soc..

[7] Liang W., Zhang B., Li L., He W. (2018). Theoretical and experimental
study on energy injection (with CO2 as an example) for modified displacement
extraction of CH4. J. China Coal Soc..

[8] Liang W., Wu D., Zhao Y. (2010). Experimental
study on CO2 displacement of coal seam
CH4. Chin. J. Rock Mech. Eng..

[9] Wu J., Ansari U. (2025). From CO2 Sequestration
to Hydrogen Storage: Further
Utilization of Depleted Gas Reservoirs. Reserv.
Sci..

[10] Su X., Song J., Guo H., Pu H., Liu X., Han Y., Zhang S., Li X., Yu S. (2020). Mechanisms and key
technologies for enhancing coal mine gas drainage productivity. Coal Sci. Technol..

[11] Liu S., Zhu C., Lin B., Liu T. (2020). Effects of spatial distribution patterns
of hydraulic slotting on pressure relief and permeability enhancement
in coal seams. J. Min. Saf. Eng..

[12] Wong S., Macdonald D., Andrei S. (2010). Conceptual economics of full
scale enhanced coalbed methane production
and CO2 storage in anthracitic coals at South Qinshui basin, Shanxi,
China. Int. J. Coal Geol..

[13] Yue G., Wang Z., Kang B. (2014). Time-varying
characteristics of the
gas diffusion coefficient of coal in low-temperature environments. China Saf. Sci. J..

[14] Yi M., Wang L., Cheng Y., Wang C., Hu B. (2022). Calculation
of gas concentration-dependent diffusion coefficient in coal particles:
Influencing mechanism of gas pressure and desorption time on diffusion
behavior. Fuel.

[15] Yang H., Wei C., Wang Z., Yang T. (2010). Numerical simulation of underground
gas injection to displace coalbed methane based on multi-physics coupling. J. China Coal Soc..

[16] Yang H., Zhang T., Wang Z., Zhao C. (2010). Experimental study
on nitrogen injection into coal seams to displace methane and promote
gas drainage. J. China Coal Soc..

[17] Wen H., Hao J., Ma L., Zheng X. (2022). Experimental Study on Replacing Coal
Seam CH_4_ with CO_2_ Gas. ACS Omega..

[18] Wen H., Fan S., Ma L., Guo J., Wei G., Hao J. (2018). Engineering
practice of promoting gas drainage in low-permeability coal seams
using low-pressure liquid CO_2_ underground. J. Xi’an Univ. Sci. Technol..

[19] Wen H., Hao J., Ma L., Ren L., Wei G., Zheng X. (2022). Study on the
adsorption characteristics of coal samples before and after liquid
CO_2_ immersion–dissolution. J. Xi’an Univ. Sci. Technol..

[20] Hao J., Wen H., Ma L., Fei J., Ren L. (2021). Theoretical Derivation
of a Prediction Model for CO_2_ Adsorption by Coal. ACS Omega.

[21] Wei G., Wen H., Deng J., Li Z., Fan S., Lei C., Liu M., Ren L. (2021). Enhanced coalbed
permeability and methane recovery
via hydraulic slotting combined with liquid CO_2_ injection. Process Saf. Environ. Prot..

[22] Sun K., Liang B., Pan Y. (2006). Study on the
productivity-enhancement
mechanism of coalbed methane extraction by gas injection under fluid–solid
coupling. Sci. Technol. Eng..

[23] Zhang W., Tai Y. (2009). Numerical simulation
of coalbed methane production by injection wells. J. Liaoning Technol. Univ. (Nat. Sci. Ed.).

[24] Wu S., Zheng A. (2007). A three-dimensional multicomponent flow model for gas-injection
displacement
of coalbed methane. Nat. Gas Geosci..

[25] Zheng A., Wang X., Liu D. (2006). Numerical
simulation study of gas-injection
displacement of coalbed methane. Pet. Drill.
Technol..

[26] Mei H., Zhang M., Li M., Sun L., Dong H., Gu H. (2004). Flow equation considering dispersion
during the gas-drive process. Nat. Gas Ind..

[27] Yang H., Feng C., Chen L. (2016). Analysis of
displacement–driving
effects and their transformation mechanisms in nitrogen injection
simulation experiments in coal seams. J. China
Coal Soc..

[28] Wang, L. Experimental study on gas-injection displacement of CH4 in deep coal seams and post-displacement characteristic traces; Ph.D. Thesis, China University of Mining and Technology: Xuzhou, China, 2013.

[29] An F., Jia H., Feng Y. (2022). Effect of stress, concentration and temperature on
gas diffusion coefficient of coal measured through a direct method
and its model application. Fuel..

[30] Wang L., Wang Z., Xu S., Zhou W., Wu J. (2015). A field investigation
of the deformation of protected coal and its application for CBM extraction
in the Qinglong coalmine in China. J. Nat. Gas
Sci. Eng..

